# Iron Bioavailability from Multiple Biofortified Foods Using an In Vitro Digestion, Caco-2 Assay for Optimizing a Cyclical Menu for a Randomized Efficacy Trial

**DOI:** 10.1093/cdn/nzab111

**Published:** 2021-09-08

**Authors:** Bryan M Gannon, Raymond P Glahn, Saurabh Mehta

**Affiliations:** Division of Nutritional Sciences, Cornell University, Ithaca, NY, USA; Institute for Nutritional Sciences, Global Health, and Technology (INSiGHT), Cornell University, Ithaca, NY, USA; USDA, Agricultural Research Service, Robert Holley Center for Agriculture and Health, Ithaca, NY, USA; Division of Nutritional Sciences, Cornell University, Ithaca, NY, USA; Institute for Nutritional Sciences, Global Health, and Technology (INSiGHT), Cornell University, Ithaca, NY, USA

**Keywords:** bioassay, biofortification, dal, food matrix, iron absorption, lentil

## Abstract

**Background:**

Inadequate nutritional status contributes to substantial losses in human health and productivity globally. A multiple biofortified food crop trial targeting iron, zinc, and vitamin A deficiencies among young children and their breastfeeding mothers is being conducted in India.

**Objective:**

We sought to determine the relative iron bioavailability from biofortified and conventional crops and crop combinations representative of a cyclical menu using crops targeted for inclusion in the feeding trial.

**Methods:**

Crops were procured from India, cooked, freeze-dried, and analyzed with an established in vitro digestion/Caco-2 iron bioavailability assay using a fixed sample weight. Crop proportions representative of meals planned for the human study were determined and combined such that samples included either all biofortified or all control crops. Crops were analyzed as single crops (*n *= 4) or crop combinations (*n *= 7) by variety (biofortified or control) in triplicate. The primary outcome was iron uptake measured by Caco-2 ferritin production normalized to total Caco-2 protein (nanograms of ferritin/milligrams of cell protein) analyzed for effects of crop variety and crop proportion using generalized linear models.

**Results:**

Biofortified pearl millet alone demonstrated higher iron uptake than conventional varieties (5.01 ± 1.66 vs. 2.17 ± 0.96; *P* = 0.036). Addition of sweet potato or sweet potato + pulse improved iron uptake for all proportions tested in control varieties and select proportions for biofortified varieties (*P* ≤ 0.05). Two multiple crop combinations demonstrated modestly higher iron uptake from biofortified crops.

**Conclusions:**

Optimizing total iron delivery should consider matrix effects, processing, and promoters/inhibitors of iron absorption in addition to total iron concentration. Future directions include evaluating recipes as prepared for consumption and comparison against human iron bioavailability studies.

## Teaser

Iron uptake from multiple biofortified crops was higher in combinations containing sweet potato or pulse relative to paired control crop combinations despite containing lower iron concentrations.

## Introduction

Inadequate nutritional status, including low intake of iron, zinc, and vitamin A, contributes to substantial health loss globally (1–3). Consequences of poor iron status alone include developmental delay, cognitive impairment, impaired physical and work performance, and adverse pregnancy outcomes (4). Inadequate status can result from poor intake, impaired bioavailability, or increased losses due to inflammation and disease states (4).

Biofortification is a strategy to enhance the nutrient content in commonly consumed food crops as a strategy to improve micronutrient status (5). Primary micronutrient targets include iron, zinc, and provitamin A due to the high burden of their deficiencies worldwide and their contributions to human health. Previous studies have demonstrated efficacy of biofortified crops in single-crop, single-micronutrient interventions, including high-iron beans, pearl millet, and rice (6–8); high-zinc maize and wheat (9); and high–provitamin A maize and sweet potato (9, 10).

Iron from plant food sources is in the form of nonheme iron. For nonheme iron to be absorbed, the iron must be solubilized and present in the reduced, ferrous state to be transported across the intestinal enterocyte (11). Nonheme iron bioavailability is known to be affected by a number of factors that can either promote or inhibit absorption. Promoters of absorption include organic acids such as ascorbic and citric acid, as well as co-consumption of meat. Common absorption inhibitors include phytates found in whole grains and pulses, oxalic acid, and polyphenols found in tea, coffee, cocoa, and some pulses. However, there is considerable variation within polyphenols; for example, some polyphenols have been shown to inhibit while others have been shown to enhance iron absorption (12, 13). When both are present, inhibitors often have more potent effects compared with promoters, resulting in a net inhibitory effect (13, 14).

In addition, there are interactions among dietary iron, the food matrix, the gut microbiome, and iron bioavailability (15). The physiological form of iron and food matrix can impact iron absorption in the upper intestine. The cotyledon cell wall is a significant barrier for iron bioavailability as it is often not broken down by cooking and human gastric and intestinal enzymes (16). Once this iron is liberated, it is likely contested for absorption with the microbiome (17).

As a result of this complex interaction between enhancers and inhibitors of iron bioavailability, not all high-iron crops or crop varieties are equally effective at delivering more bioavailable iron. For example, high-iron carioca beans were shown to be efficacious in increasing hemoglobin concentrations when consumed along with representative diet-based feeds in chickens (18, 19), and studies have also shown that varieties of the yellow bean market class deliver better iron for absorption (20).

A multiple biofortified food crop trial was recently completed in southern India (Clinicaltrials.gov NCT02648893) (21); the work described in this article was completed during the planning stages of this trial. The study included young children in the complementary feeding period and their mothers. Two intervention arms were used to evaluate the impact of recipes prepared with multiple biofortified crops compared with similar recipes prepared with control crops on health outcomes including biomarkers of nutritional status. The crops evaluated in this study include pearl millet (high iron and zinc), wheat (high zinc), sweet potato (high provitamin A), and pulses (high iron and zinc). Recipes, meals, and a cyclic menu for the human feeding trial were formulated by combining multiple crops in traditional and novel recipes to balance delivery of iron, zinc, and vitamin A over the course of each day and week. Initial recipes were shown to be highly acceptable among children and mothers in the study population (22).

Single-crop iron bioavailability from pearl millet, sweet potato, and pulse has been assessed using human, animal, and in vitro models. Biofortified pearl millet showed similar iron bioavailability as conventional pearl millet at ∼7.5%, resulting in more absorbed iron from biofortified varieties in humans (23) and improved iron status in chickens (24). Pulses, including lentils, contain substantial iron concentrations. However, human studies have shown this iron has low bioavailability (25, 26) and it is inversely related to iron status (25). Carotenoids, including β-carotene, have been shown to be beneficial for iron bioavailability from cereal-based meals (27) and complementary foods with orange sweet potato (28). Different sweet potato lines vary in their promotion or inhibition of iron uptake, attributed to ascorbic acid, chlorogenic acid, and polyphenols (29).

Due to the complex interactions among crop combinations, crop varieties, and promoters/inhibitors of iron bioavailability, we sought to estimate the relative iron uptake from crop combinations corresponding to acceptable meals to determine which crops and crop combinations would be most favorable for delivering iron to include in the human efficacy trial. We used an established, in vitro digestion/Caco-2 screening tool for iron bioavailability (30, 31) that has demonstrated concordance with common promoters and inhibitors of iron uptake (32).

## Methods

### Reagents

All reagents were purchased from Sigma Chemical Co. (St. Louis, MO) unless stated otherwise.

### Crops

Crops used in this study correspond to those selected for inclusion in a human efficacy study. For this study, initial convenient 500-g samples were collected for each crop and variety. Biofortified varieties contain higher concentrations of micronutrients, including high-zinc wheat, high-iron and zinc pearl millet, and high–provitamin A (β-carotene) sweet potato. Wheat and pearl millet were coordinated and grown with oversight by the International Crops Research Institute for the Semi-Arid Tropics (ICRISAT) and milled into whole-grain flour. Wheat varieties used were biofortified, BHU-6, and control, HD2967. Pearl millet varieties used were biofortified, Dhanashakti, and control, DG9444. Pulse varieties used were a locally available, high-iron variety, red lentil (*Lens culinaris*, Masoor dal), and a locally available control, pigeon pea (*Cajanus cajan*, Toor dal). Locally available high-iron red lentil was paired with the biofortified crops in this study and reported with biofortified crops for convenience. Pigeon pea was chosen as the control pulse because it is consumed locally, is included in school feeding programs, and is comparatively lower in iron than lentils chosen for the efficacy trial. Pulses were dehulled before use. Sweet potato was coordinated and grown with oversight by the International Potato Center (CIP). Sweet potato varieties were biofortified, Kamalasundari, and control, white local variety. Wheat, pearl millet, and pulses were stored in a commercial climate-controlled storage facility (53–56% humidity; temperature: 6–14°C).

### Nutrient concentration of crops

Mineral concentrations of crops were determined in triplicate by inductively coupled plasma–atomic emission spectrometry (ICP-AES), similarly as published (30). Briefly, 0.5 g of dried, ground samples were digested with 60:40 HNO_3_:HClO_4_, diluted to 20 mL, and analyzed with a Thermo iCAP 6500 series (Thermo Jarrell Ash Corp.). Sweet potato β-carotene concentrations were determined in quadruplicate using HPLC, method AOAC 2016.13 (33), by SGS India.

### Crop cooking

All crops were cooked, freeze-dried, and ground before use according to established laboratory protocols (pearl millet, pulses) or as used in the feeding trial (sweet potato, wheat) to standardize heat and moisture exposure. All water used was 18 MΩ.

Sweet potatoes were washed, steamed for ∼20 min, peeled, cut, puréed, vacuum-sealed, and frozen at −18°C. Wheat was prepared as a *chapati*: wheat flour was combined with water to form a dough, which was pressed and cooked in a ceramic pan for ∼5 min.

Pearl millet (∼60 g, weighed) was rinsed 3 times with water, and 180 mL water was added. Pearl millet and water were soaked at room temperature for 15 min and then boiled for 60 min, allowed to cool, and frozen together with cooking water. Pulses were hand-cleaned to remove any differing in visual appearance, weighed (∼60 g), rinsed 3 times with water, and 180 mL of water was added. Pulses and water were soaked at room temperature for 15 min and then were boiled for 15 minutes, allowed to cool, and frozen together with cooking water.

### Crop combinations

Crop combinations for this study were structured to correspond to meals composed of single or multiple recipes as would be consumed at a single sitting (22). Briefly, traditional or novel recipes incorporating crops of interest were structured into a 2-wk cyclical menu. The cyclical menu used variations of single or multiple recipe combinations for breakfast, lunch, and snack for 6 d/wk. Recipes were prepared identically using either all biofortified or all control crops; locally available high-iron red lentil was included with biofortified crops and locally available pigeon pea was included with control crops.

**FIGURE 1:**
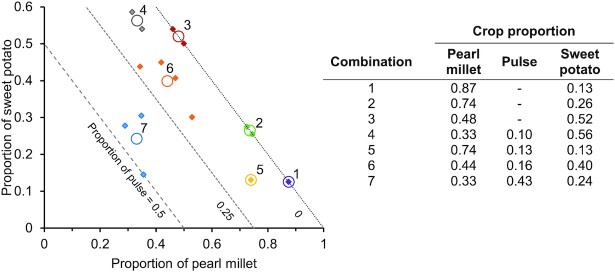
Crop proportions used for the iron bioavailability assay. K-mean centroids (open circles) were used to represent summary estimates of meals with similar crop proportions (solid diamonds). Dashed contour lines indicate pulse proportion. The proportions of pearl millet, sweet potato, and pulse sum to 1 for each individual meal and cluster.

Crop proportions for 15 meals were determined from a planned cyclical menu. Since some planned meals contained similar crop proportions, we sought to identify a reduced set of representative crop combinations to analyze for iron bioavailability. K-means clustering was used to identify 7 clusters of recipes with similar crop proportions and 7 corresponding centroids as summary estimates for each cluster that were used for crop combinations in this study (**Figure 1**). The primary crops delivering iron (pearl millet, pulse, and wheat) were also analyzed as single crops.

Sweet potato water content differed by variety. Therefore, sweet potato proportions were determined using wet weight as prepared and consumed in the efficacy study, and a dry/wet-weight correction factor was applied differentially to orange (0.20) and white (0.31) sweet potato as samples were freeze-dried for the assay. All samples totaled 0.5 g adjusting for water content of sweet potato. Samples were weighed and analyzed in triplicate for each crop combination and variety, except for single-crop pearl millet and pulse samples, both of which had 6 replicates.

### In vitro digestion, Caco-2 iron bioavailability assay

The in vitro digestion, Caco-2 iron bioavailability assay was conducted as previously described (30, 31). For each crop variety, each crop or crop combination was digested and analyzed in triplicate. Single-crop pearl millet and pulse sample size was *n*  = 6. Briefly, the samples begin with a gastric phase that included pepsin at pH 2 with rocking for 1 h at 37°C. Then, the sample pH was raised to 5.5–6, pancreatin-bile extract solution was added, the pH was adjusted to ∼7.0, the volume was adjusted to 15.0 mL, and samples were incubated for 2 h at 37°C. This sample digest was then used for the Caco-2 assay.

The Caco-2 assay consists of 6-well culture plates containing 2-chamber wells with a dialysis membrane separating the upper well from the lower well containing the Caco-2 cell monolayer. The same cell passage was used on all plates. Sample replicates were split across different plates. A 1.5-mL aliquot of the digest was transferred to the upper well, allowing soluble iron to pass through the membrane during rocking (6 oscillations/min for 120 min). The upper well was then removed, and cells were allowed to incubate for a further 22 h. Cells were then harvested for determination of ferritin (FER-IRON II Ferritin Assay; RAMCO Laboratories) and total protein determination with a semi-micro adaptation of the Bio-Rad DC protein assay kit (Bio-Rad Laboratories).

### Cell imaging

Cells were imaged to assess cotyledon cell-wall integrity due to its influence for iron bioavailability. Freeze-dried crops (100 mg) were suspended in 2.9 mL buffered saline (130 mM NaCl, 5 mM KCl, 5 mM Pipes buffer, pH 6.7). The solution was carefully mixed, and 100 µL was added to 1.4 mL of saline containing 50 µL trypan blue solution (0.4% trypan blue in 0.9% saline) for a final dilution factor of 450. This suspension was dropped (20 µL) on glass slides with no cover slip and visualized with a Nikon Stereo Microscope (Model SMZ1500; Nikon Instruments, Inc.) coupled to a Nikon DS-Fi1c LV-TV imaging system at 56.25× magnification.

### Data analysis

Statistical analysis was done in SAS version 9.4 (SAS Institute) using generalized linear models or RStudio [Version 1.1.456; RStudio Team (2020). RStudio: Integrated Development for R. RStudio, PBC, Boston, MA; http://www.rstudio.com/] for k-means clustering. Iron uptake was calculated as Caco-2 ferritin normalized to Caco-2 total protein. Relative iron uptake was calculated as iron uptake normalized to sample iron content. For single-crop comparisons, the effect of crop variety within crop was determined for each crop. For multiple-crop comparisons, the effects of crop proportions were assessed within crop variety, and the effect of crop variety was assessed within crop proportions; effects are presented separately. Residual normality and homogeneity of variance was confirmed; and if assumptions were violated, variables were transformed before analysis. *P* < 0.05 was considered significant. Post hoc letter groupings were determined using Tukey-adjusted differences.

## Results

### Crop nutrient concentrations

Crop iron, zinc, and β-carotene concentrations are presented in **Table 1**. Biofortified pearl millet, wheat, sweet potato, and pulse had higher iron concentrations than conventional counterparts. The greatest within-crop iron differentials were with pearl millet (38.5 µg/g) and pulses (46.0 µg/g), approximately double the iron concentration between biofortified and control varieties. Wheat and sweet potato were ∼20–30% higher in iron than control varieties. Zinc concentrations were also higher in biofortified crop varieties, except for sweet potato. Biofortified pearl millet and wheat both contained ∼50–60% higher zinc concentrations than control varieties. Biofortified orange sweet potato contained substantial β-carotene, while control white sweet potato contained very low concentrations.

**TABLE 1:** Crops used with nutrient concentrations^1^

	Crop nutrient concentration, µg/g							
	Pearl millet		Wheat		Sweet potato		Pulse	
	Biofortified	Control	Biofortified	Control	Biofortified	Control	Biofortified	Control
Iron	77.6 ± 1.7	39.1 ± 0.6	61.2 ± 8.1	47.5 ± 0.3	6.7 ± 0.1	5.7 ± 0.3	62.1 ± 0.6	30.2 ± 0.4
Zinc	53.8 ± 0.3	33.8 ± 0.5	46.4 ± 5.6	30.1 ± 0.3	0.9 ± 0.01	1.2 ± 0.02	31.9 ± 0.2	25.1 ± 0.2
β-Carotene	—	—	—	—	66.1 ± 6.8	0.26	—	—

1 Values are means ± SDs; *n *= 3 (sweet potato β-carotene, *n *= 4). Sweet potato mineral concentrations are reported on a wet weight basis. Dry matter proportion for sweet potato was biofortified: 0.20, control: 0.31.

### Single-crop iron uptake

Iron uptakes from single crops of pearl millet, wheat, and pulses are presented in **Table 2**. Biofortified pearl millet resulted in higher iron uptake than control pearl millet. Red lentil resulted in higher uptake than pigeon pea. Biofortified and control wheat had similar iron uptake.

**TABLE 2:** Single-crop iron uptake^1^

	ng Caco-2 ferritin/mg Caco-2 protein		
	Pearl millet	Pulse	Wheat
Biofortified	5.01 ± 1.66	9.58 ± 1.61	4.64 ± 1.13
Control	2.17 ± 0.96	4.26 ± 0.61	5.37 ± 1.13
*P* value^2^	0.036	0.0011	0.47

1 Values are means ± SDs; each value represents *n *= 3 for each crop and variety. Pearl millet and pulse ferritin values are reported as a subset (*n *= 3) to facilitate comparisons with crop combinations from the same experiment; statistical analysis included all data (*n* = 6).

2 *P* value testing the null hypothesis of no difference by crop variety (biofortified vs. control).

### Multiple-crop iron uptake

#### Among control crops

**FIGURE 2:**
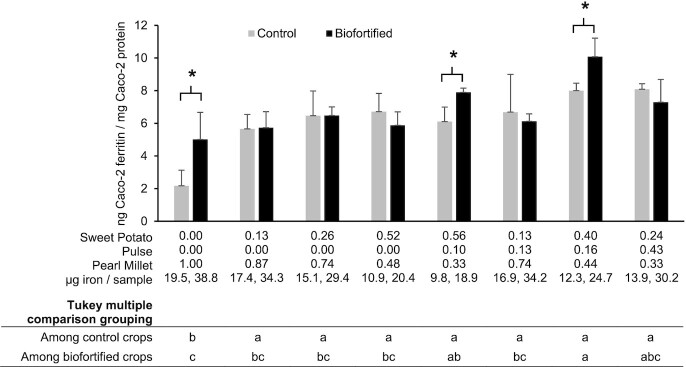
Iron uptake (nanograms of cell ferritin/milligrams of cell protein) within and between control and biofortified crop combinations. Crop proportions are indicated for each recipe. Values are means ± SDs; *n *= 3. Comparisons with an asterisk (*) are significantly different between crop varieties within recipe. Within control or biofortified varieties, combinations without a common letter are significantly different from each other.

For comparisons among control crop combinations of pearl millet, sweet potato, and pigeon pea, pearl millet alone had the lowest amount of iron uptake despite having the highest sample iron concentration (**Figure 2**). All 7 other crop combinations including either sweet potato or sweet potato and pigeon pea had significantly higher iron uptake but did not differ among these multi-crop combinations.

#### Among biofortified crops

For comparisons among biofortified crop combinations, pearl millet alone did not differ in iron uptake than a number of multi-crop combinations (Figure 2). Two combinations of crops—(33%, 10%, 56%) and (44%, 16%, 40%) pearl millet, pulse, and sweet potato, respectively—demonstrated elevated iron uptake relative to pearl millet alone and some multi-crop combinations.

#### Between biofortified and control crops

For comparisons between biofortified and control combinations, 3 combinations demonstrated statistically higher iron uptake from biofortified crops, with the remaining combinations demonstrating no difference in iron uptake (Figure 2). These combinations were 100%, 0%, 0%; 33%, 10%, 56%; and 44%, 16%, 40% pearl millet, pulse, and sweet potato, respectively.

### Relative iron uptake

**FIGURE 3:**
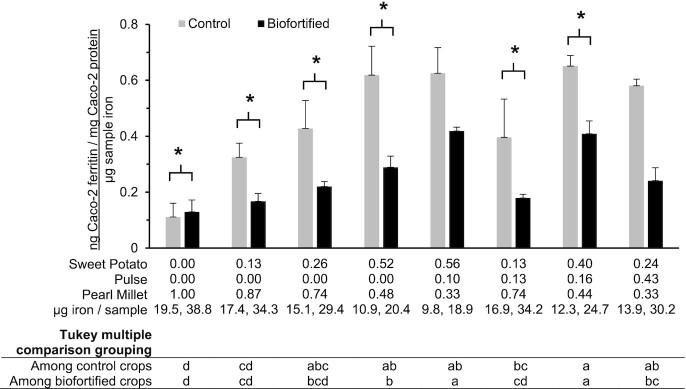
Relative iron uptake (nanograms of cell ferritin/milligrams of cell protein/micrograms of sample iron) within and between control and biofortified crop combinations. Crop proportions are indicated for each recipe. Values are means ± SDs; *n *= 3. Comparisons with an asterisk (*) are significantly different between crop varieties within recipe. Within control or biofortified varieties, combinations without a common letter are significantly different from each other.

Iron uptake normalized to iron per sample is presented in **Figure 3**. Relative iron uptake was increased with the addition of both sweet potato and pulse in both control and biofortified varieties. Most crop combinations had higher relative iron uptake for control crops relative to biofortified crops.

### Cell imaging

**FIGURE 4:**
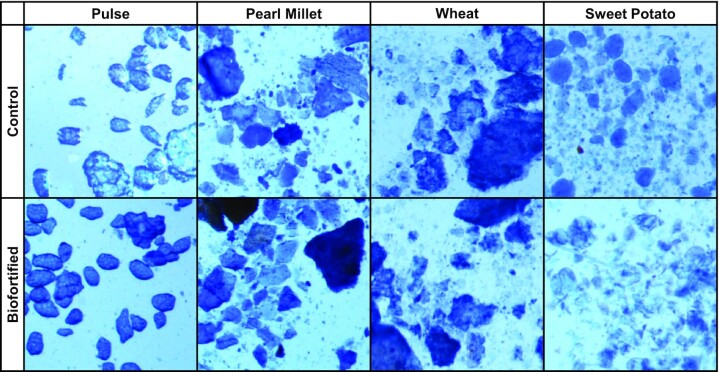
Representative images of crop cells after cooking and prior to any digestion. Cooked crops were suspended in saline, stained with trypan blue, and imaged at 56.25× magnification. Biofortified and control pulse varieties are noted to have intact cotyledon cell walls. Pearl millet and wheat did not demonstrate any significant cellular structure and reflected cellular debris of varying size. Sweet potato showed a combination of starch granules and broken cell material, with control sweet potato containing more defined and larger starch granules.

Representative cell images are presented in **Figure 4**. Both biofortified and control pulse varieties are noted to have intact cotyledon cell walls. Pearl millet and wheat did not demonstrate any significant cellular structure and reflected cellular debris of varying size. Sweet potato showed a combination of starch granules and broken cell material, with control sweet potato containing more defined and larger starch granules.

## Discussion

This study determined relative iron uptake of combinations of multiple biofortified or control crops based on representative meals to be used in a human feeding trial. Two primary findings from this study were as follows: *1*) in both biofortified and control crops, the addition of sweet potato and pulse improved iron uptake despite having lower iron concentrations, and *2*) some biofortified crop combinations provided higher iron uptake relative to control crop combinations.

Nutrient bioavailability is a critical component linking targeted intakes with absorbed nutrient and resulting physiological function and health outcomes. If bioavailability remains constant, consuming foods with higher iron concentrations will result in more absorbed iron. However, bioavailability is not necessarily constant as dietary intakes increase, either due to *1*) homeostatic regulation of absorption or *2*) correlations of higher iron foods with higher nutrients competing for absorption (e.g., iron and zinc) or inhibitors of absorption (e.g., phytate or polyphenols), resulting in lower bioavailability, yet similar absorbed nutrient. As shown in this study, iron uptake can be increased by combining strategies of iron biofortification to increase iron intakes with food matrix combinations to ensure bioavailability. Bioavailability can be a separately targeted trait to optimize absorbed nutrient at multiple stages of food production, processing, preparation, and consumption.

Further determination of the promotion or inhibition of iron absorption by the food matrix and interactions among foods can provide other complementary targets and a multipronged approach to improve total iron delivery. This approach may further mitigate any adverse outcomes from having excess unabsorbed iron being provided to the gut microbiome (15). While this study focused on iron, addition of other crops (e.g., sweet potato) increased iron uptake, and can also serve as a significant source of provitamin A, which is known to synergize with iron and zinc interventions (27, 28, 34).

In this study, pearl millet alone had relatively low bioavailability compared with crop combinations, and biofortified pearl millet demonstrated increased iron uptake relative to conventional pearl millet. This agrees with previous research demonstrating improved iron delivery with biofortification despite low bioavailability, which is similar between crop varieties (23, 24). High-iron pearl millet was shown to improve iron status in chickens, although relatively low values observed for in vitro iron absorption were likely due to substantial inhibitors present in pearl millet (24). Biofortified pearl millet showed similar bioavailability as conventional pearl millet at ∼7.5%, resulting in more absorbed iron from biofortified varieties in humans (23). Iron-biofortified pearl millet also improved biomarkers of iron status in adolescents, particularly among those who were iron deficient (6).

Pulse iron uptake has been shown to be low in humans (25, 26). This has been attributed to inhibitors of iron absorption and the food matrix. The seed coat contains absorption inhibitors including phytates and polyphenols, and removal of the seed coat has been shown to improve lentil iron bioavailability (35). In addition, cell walls being intact prior to digestion is known to reduce iron bioavailability (16). Pulses used in this study were dehulled before analysis, and we also observed that pulse cell walls were intact prior to digestion, resulting in a net unknown effect on iron uptake from pulses in this study. We observed that pulses evaluated alone or as part of crop combinations performed well compared with crop combinations without pulse, indicating a net favorable contribution to iron uptake from dehulled pulses used in this study.

The favorable iron uptake from pulses used in this study may be due to specific varieties of pulse that were used for this study, or potentially the lack of inhibitors that may have been present in test meals used for human studies. For example, other components in human test meals may potentially influence iron bioavailability, including promotion by garlic and onions (36), mixed effects from turmeric/curcumin (37–39), and unknown resulting effects of flavonoids and phenolic compounds in bay leaf (40).

Enhanced iron uptake was seen in both varieties of sweet potatoes, despite reduced total iron available in these samples due to lower iron concentrations in the sweet potato than pearl millet or pulses. Different sweet potato lines can vary in their promotion or inhibition of iron uptake, attributed to relatively low phytate compared with cereals and considerable ascorbic acid, chlorogenic acid, and polyphenols (29, 41).

Sweet potato digestion products, primarily polyphenols, have been shown to inhibit uptake of supplemental FeCl_3_ with ascorbic acid in a Caco-2 model (42). Carotenoids, including β-carotene, have been shown to be beneficial for iron bioavailability from cereal-based meals (27) and complementary foods with orange sweet potato (28). Sweet potato improved zinc bioavailability from sorghum or maize porridges; however, iron uptake was not impacted by the addition of sweet potato in an in vitro model (34). Total iron absorption from high-iron orange-fleshed sweet potatoes was higher than regular orange-fleshed sweet potatoes in women in Malawi (43).

In addition to increased iron uptake with the addition of sweet potato in both biofortified and control crops, we observed that 2 out of 7 tested biofortified crop combinations demonstrated higher iron uptake compared with control varieties. While the iron content of the biofortified samples was consistently higher, it is possible that the orange sweet potato could be having an additional enhancing effect on iron bioavailability in this context of multiple biofortified crops. Future studies could specifically evaluate the effect of different potato types and components, including provitamin A sweet potato, to investigate this interaction while also targeting multiple micronutrients.

It is challenging to predict and optimize iron uptake from meals with the multilevel variability and interactions among crops, crop varieties, and the complex interactions among iron concentrations, enhancers, inhibitors, and competitors of absorption. Therefore, utilization of relatively inexpensive in vitro and in vivo screening tools thus represents a cost-effective approach to determine if an iron-biofortified crop variety holds promise to deliver more iron for both their identification as well as for planning menus in human efficacy trials.

Strengths of this study included the use of a validated iron bioavailability assay, using the same crops being used in a human feeding trial, and using machine-learning techniques to cluster similar meals. The in vitro digestion/Caco-2 iron bioavailability assay used has been thoroughly validated and demonstrated concordance with human feeding trials (6, 12, 16, 19, 24, 44). This methodology has also been used to evaluate Bangladeshi meal plans to determine factors related to iron bioavailability in combinations representing meals commonly consumed (45). Crops used for this study are the same as used for a recently completed human feeding trial (Clinicaltrials.gov NCT02648893). Further, crop proportions mimic recipes and combinations that represent common combinations of crop proportions for meals delivered to study participants. K-means clustering was used to cluster similar meals based on their crop proportions in order to more efficiently test the spectrum of meals used in the trial.

Limitations of this study included that simulated recipes were based on proportions of crops of interest, instead of the individual recipe prepared as it would be consumed. As a first step in investigating total bioavailable iron from multiple biofortified crops, this study provides data on crop components alone. Subsequent experiments can determine the bioavailability of recipe samples, and account for extra inhibitors/promoters of absorption, particularly of prepared recipes as these contain additional ingredients that may impact the iron bioavailability of supplied meals (e.g., fruits, vegetables, spices). Crops were selected primarily for concentrations of micronutrients of interest; total iron delivery for biofortified crops could still potentially be enhanced by targeting bioavailability in addition to micronutrient concentrations throughout the breeding and evaluation pipeline. Further, our study compared all biofortified crops with all control crops and is therefore unable to provide evidence on specific crop interactions—for example, with β-carotene–containing sweet potato.

While some biofortified crop combinations demonstrated higher iron uptake than control crops, we saw larger effects on iron uptake due to the combination of crops rather than iron concentration alone. This indicates that there are additional avenues by which iron status may be improved, including targeting dietary diversity, bioavailability, and food accessibility and affordability (46). Dietary diversification or modification strategies provide multiple avenues to increase total absorbed iron or zinc (47), and it has been suggested that a range of meal composition and human factors (e.g., iron status, obesity) be included when estimating iron bioavailability (48). There are ongoing efforts to test the addition of vitamin C–rich fruit to regular meals in order to address iron deficiency anemia (49).

Because we did not observe crop combinations with lower iron uptake than pearl millet alone, we concluded that our recipes and meals were designed and combined in a way that would provide absorbable iron while also being acceptable to participants. Remaining research areas include further evaluation of additional recipe ingredients and the resulting enhancement or inhibition of iron bioavailability and determination of the impact of long-term consumption of biofortified crop combinations on growth, nutritional status, and health outcomes in humans.

## Notes

Supported by the USDA, Agricultural Research Service Project 8062-52000-001-00-D (RPG). The work was also supported by HarvestPlus grant number 2015H8336 (SM) awarded to Cornell University, in part by the Bill & Melinda Gates Foundation through Grant OPP1019962 to HarvestPlus. HarvestPlus’ principal donors are the UK Government; the Bill & Melinda Gates Foundation; the US Government’s Feed the Future initiative; the Government of Canada; the European Commission; and donors to the CGIAR Research Program on Agriculture for Nutrition and Health (A4NH). HarvestPlus is also supported by the John D. and Catherine T. MacArthur Foundation. HarvestPlus India supplied crops used in this study.

Author disclosures: SM has an equity interest in VitaMe Technologies (DBA VitaScan), which is commercializing technology focused on developing point-of-care assays for nutritional status based on his research as a faculty member at Cornell University. The other authors report no conflicts of interest.

The funders had no role in the design of the study; in the collection, analyses, or interpretation of data; in the writing of the manuscript; or in the decision to publish the results.
